# Medicinally active principles analysis of *Tephrosia apollinea* (Delile) DC. growing in the United Arab Emirates

**DOI:** 10.1186/s13104-017-2388-0

**Published:** 2017-01-25

**Authors:** Abdul J. Cheruth, Saif A. M. Al Baloushi, Kandhan Karthishwaran, Sajid Maqsood, Shyam S. Kurup, Sabitha Sakkir

**Affiliations:** 1 Department of Aridland Agriculture, College of Food and Agriculture, United Arab Emirates University, P.O. Box 15551, Al Ain, United Arab Emirates; 2 Department of Food Science, College of Food and Agriculture, United Arab Emirates University, 15551, Al Ain, United Arab Emirates; 3 Terrestrial and Marine Biodiversity Sector, Wildlife Assessment and Conservation, Environment Agency-Abu Dhabi, P.O. Box 45553, Abu Dhabi, United Arab Emirates

**Keywords:** Phytochemical, *Tephrosia apollinea*, Proximate, Antioxidant, Free radical

## Abstract

**Background:**

*Tephrosia apollinea* is a leguminous plant and is native to southwest Asia, Arabia, northwestern India and northeast Africa. In traditional system, it is used for medicinal and coloring purpose. The present study aims to explore the phytochemical, proximate analysis, element contents and antioxidant potential of *T. apollinea* extract.

**Methods:**

The phytochemical screening was done with qualitative methods. Proximate analysis and elemental composition were performed from powdered sample. In vitro antioxidant assays such as 1,1-diphenyl-2-picrylhydrazyl (DPPH) radicals and reducing power-scavenging assays were used for evaluating the antioxidant properties.

**Results:**

Qualitative screening of methanolic extract of *T. apollinea* showed the presence of alkaloids, phenolics, flavonoids, terpenoids, glycosides and saponins. The methanolic extract of *T. apollinea* exhibited a significant dose dependent inhibition of DPPH activity, with a 50% inhibition (IC^50^) at a concentration found to be 29.41 µg/ml, which was compared with standard GAE (IC^50^ = 31.09 μg/ml). The reducing power shows good linear relationship in both standard gallic acid (R^2^ = 0.956) and *T. apollinea* extract (0.984).

**Conclusions:**

The results of our study clearly suggested that the methanolic extract of *T. apollinea* may serve as potential source of natural antioxidant for nutraceutical application.

## Background

Traditional medicinal system of United Arab Emirates utilizes many native plants, and increasing amounts of evidence has revealed the presence of potent antioxidant activity in herbal extracts [1, 2]. Crude extracts of UAE plants showed many medicinal properties including antioxidant effects, so there will be vast potential of medicinal plants as source of new drugs [2, 3]. Scientific evidence supports that rationale of using native plants and traditional formulations in health care. In this modern era, all medical systems rely on synthetic medicine, but the plant materials remain an important resource for combating illnesses, including infectious diseases. Out of the native plants used in traditional medical systems, many have been investigated for potential drugs, alternative medicine, food additives, agrochemicals and industrial chemicals [4].

According to World Health Organization (WHO) 80% of the population of developing countries still relies on traditional medicines, mostly plant derived drugs, for their primary health care needs [5, 6]. Phytochemicals are naturally occurring bioactive compounds present in plants, which acts as agents for protection of tissues from stress, diseases and other deleterious effects. Phytochemicals can be primary or secondary metabolites, they may be pigments, proteins, sugars, terpenoid, alkaloids and phenolic compounds [7]. Medicinal plants are a major natural alternative to synthetic drugs, and nowadays, native plant usage in traditional as well as modern medicine is gaining a lot of attention [8].

*Tephrosia apollinea* (Delile) DC. is a perennial shrub and legume species, and one of the most common plants in the lower mountains of the UAE [9]. There are many traditional medical uses for this plant, e.g. leaves for relieving earache and pain from fractures, bark for removing ticks from camel ears [9]. The plant possesses insecticidal and anti-cancer properties [10, 11]. The plant possesses medicinal properties and has significant anti-bacterial properties; the leaves and the root have been used to treat bronchitis, cough, earache, wounds and bone fractures by herbalists in countries like Oman [9, 12].

*Tephrosia purpurea* extracts showed the presence of isoflavones, flavanones, flavanols and flavones [13]. The leaves of *Tephrosia* contains semiglabrin, semigalbrinol, and a flavanone named apollineanin [14]. Recently, there are reports indicating that a number of species of *Tephrosia* possess medicinal properties [15]. These studies help in identification and scientific validation of *Tephrosia*. Reactive oxygen species (ROS) are responsible for many diseases of cardiovascular systems, neurodegenerative disorders, diabetes mellitus and cancer. Antioxidants obtained from natural resources gained high research interest to face diseases generated by ROS [16]. In this regard, it seems important to estimate the natural antioxidant and phytochemical contents of native plants used in the traditional medical systems of UAE. This study aims to investigate the scientific basis for the use of *T. apollinea* plant by analyzing the phytochemical constituents, proximate and mineral compositions and the free radical scavenging activity from the aerial plant parts.

## Methods

### Plant collection

The fully matured plants of *T. apollinea* were collected from Al-Foah Experimental Station of College of Food and Agriculture, United Arab Emirates University and from Experimental Nursery of Terrestrial and Marine Biodiversity Sector, Wildlife Assessment and Conservation, Environment Agency (EAD)-Abu Dhabi, UAE. The plants were identified and authenticated at UAEU and EAD. For the plant samples, voucher specimens were deposited at the COS-UAEU Herbarium located in the Department of Biology (Lab E-3), College of Science at the UAEU, United Arab Emirates.

### Preparation of extracts

Collected plant material was thoroughly washed and dried in the shade at 25 ± 2 °C for about 10 days. Samples were powdered and stored in airtight containers at room temperature. The powdered materials of the plant species (500 g) were soaked in 1.5 l of methanol (Sigma-Aldrich, USA) for 1 day, followed by Soxhlet extraction by using methanol for 72 h. At the end of extraction, it was passed through Whatman filter paper No.1 (Whatman Ltd., England). The extract was concentrated to dryness under vacuum on rotary evaporator at 40 °C then stored at 4 °C for further use.

### Tests for phytochemicals

Phytochemical tests were done on the methanolic extract using standard qualitative methods as previously described [17–19] for the analysis of flavonoid, carbohydrate, alkaloid, saponin, phenol, tannin, phlobatannins, terpenoids, cardiac glycosides, proteins and volatile oils. These tests gives only the presence or absence of the tested parameters.

### Total phenol and flavonoid estimation

Quantification of total phenolic content in methanolic plant extract of *T. apollinea* were done by the Folin–Ciocalteau reagent method [20]. Total phenolic compounds contents of the extracts was determined as mg of gallic acid equivalent (GAE) by using standard equation, which obtained from the standard gallic acid curve. Total flavonoids in the plant was determined by the method of Zhishen et al. [21]. The total flavonoid content in the extract was expressed as mg quercetin equivalents (QE).

### Proximate analysis and elements estimation

Dry matter, moisture, crude protein, fibre, fat, ash and carbohydrate contents were determined by standard methods of the Association of Official Analytical Chemists (A.O.A.C) [22–25]. The elemental analysis was done by the standard method (Method 3015A, US Environmental Protection Agency, 2008) and as explained previously [26].

### Antioxidant analysis

Antioxidant activity was analyzed by using 2, 2-diphenyl-1-picrylhydrazyl (DPPH) method [27] and Reducing power assay [28].

### Statistical analysis

All the experiments were carried out in triplicate, and the results were expressed as mean ± SD. Statistical analysis was performed using SPSS 13.0 and Excel 2003.

## Results

Table 1 shows the results of the phytochemical analysis of the plant parts of *T. apollinea*. There are various secondary metabolites of therapeutical importance. Out of them, major phytochemicals were phenols, saponins, tannins, flavonoids, terpenoids, phlobatannin and alkaloids. However, the extract tested showed the absence of cardiac glycoside.

**Table 1:** The analysis of phytochemicals in the methanol extract of *T. apollinea*

Phytochemical constituents	Observation	Inference/results
Flavonoids	Yellow colour persist	+
Carbohydrates	Green colour	+
Alkaloids	Orange precipitate	+
Saponin	Formation of emulsion	+
Phenols	Blue color	+
Tannins	Green brownish color	+
Phobatannins	Red precipitate	+
Terpenoids	Reddish brown colour	+
Cardiac glycosides	No yellowish brown ring of upper layer	–
Proteins	White precipitate which turns red	+
Volatile oils	White precipitate	+

*+* presence, *−* absence

Total phenolics and flavonoids contents of methanolic extract of *T. apollinea* were 12.36 mg GAE/g and 4.18 mg QE/g respectively.

Proximate compositions of the aerial plant parts are given in Table 2. Proximate compositions were done from dry basis and expressed in percentage (%). The fibre and ash contents were high and suggested the high nutritive value of *T. apollinea*. The carbohydrate, protein and moisture contents were found in appreciable amounts.

**Table 2:** Proximate composition of aerial parts of *T. apollinea* (g/100 g) of dried sample

Parameter	Concentration (dry weight basis)
Dry matter	89.47
Moisture%	10.53
Crude protein% DM	16.41
Fibre%	39.44
Fat (EE%)	6.83
Ash	8.15
Carbohydrate	18.64

Nutritional composition was analysed on basis of micro and macro elemental analysis. The mineral compositions of the plant samples were presented in Table 3. Fe, Ca, Mg, Na and Zn were present in appreciable quantities. Low concentrations of phosphorous, copper and potassium were observed in *T. apollinea*.

**Table 3:** Mineral composition of *T. apollinea*

Microelements (mg/Kg)	
Zn	41.2
Cu	6.695
Cr	3.23966
Fe	273.6
Pb	<0.011
Mn	18.3
Ni	5.05
Cd	4.389
Co	0.172
Macroelements (mg/Kg)	
Ca	1.525
Na	3.57
K	1.076
Mg	2.712
S	2.825
P	2.15

The reactivity of the test compounds with a stable free radical was evaluated by DPPH scavenging assay. DPPH gives a strong absorption band at 517 nm in visible region. The results (Table 4) revealed DPPH radical-scavenging activity of the methanol extracts of the *T. apollinea*. It elucidates the mean values across the concentration range, indicating that the methanol extracts of *T. apollinea* are more potent in scavenging the DPPH radicals generated in vitro, when compared to the standard GAE. The methanolic extract of *T. apollinea* exhibited a significant dose dependent inhibition of DPPH activity, with a 50% inhibition (IC^50^) at a concentration found to be 29.41 µg/ml, which was compared with GAE (IC50 = 31.09 μg/ml). This result demonstrated that *T. apollinea* methanolic extract has inhibitory activity against the DPPH radical.

**Table 4:** DPPH radical scavenging activity and reducing power of methanolic extracts of *T. apollinea* in comparison with gallic acid

Antioxidant activity					
Concentration (µg/ml)	Inhibition (%)		Concentration (µg/ml)	Absorbance 700 nm	
	GAE	TAE		GAE	TAE
10	38.1 ± 1.8	40.6 ± 2.2	10	0.061 ± 0.015	0.268 ± 0.024
20	43.1 ± 2.7	54.6 ± 1.8	20	0.072 ± 0.022	0.298 ± 0.028
30	58.1 ± 1.2	59.6 ± 3.8	30	0.084 ± 0.017	0.314 ± 0.022
40	62.1 ± 3.4	61.5 ± 2.6	40	0.091 ± 0.013	0.333 ± 0.031
50	67.5 ± 4.3	72.1 ± 3.3	50	0.110 ± 0.011	0.340 ± 0.024
IC50	29.41	31.09	R^2^ value	0.956	0.984

Values are the average of triplicate experiments and represented as mean ± standard deviation

*GAE* gallic acid equivalent, *TAE T. apollinea* extract

The reducing power of extract is given in Table 4, at each concentration, in the range of 10–50 µg/ml compared to GAE. The methanolic extract of *T. apollinea* exhibited a significant dose dependent inhibition of reducing power-scavenging activity. The reducing power of extract of *T. apollinea* was very potent and the reducing power of the extract and was increased with quantity of sample. The plant extract could reduce the most Fe^3+^ ions, which had a lesser reductive activity than the standard of GAE. The reducing power shows good linear relationship in both standard gallic acid (R^2^ = 0.956) and *T. apollinea* extract (0.984). Therefore, ferric reducing antioxidant activities of methanol extract of *T. apollinea* indicating the ability of plant extract to reduce Fe^3+^ to Fe^2+.^ The methanolic extract of *T. apollinea* exhibited a significant dose dependent inhibition of reducing power activity.

## Discussion

Medicinal plants are a pool of drugs of traditional systems of medicine, also modern medicines relies a lot in traditional plants. United Arab Emirates has a wide variety of medicinal plants in its relatively desert and arid flora [1–3]. Many synthetic drugs are being replaced by herbal plants due to their nutraceuticals values, and are often without side effects [29]. The medicinal and nutritional potentials of the aerial parts of *T. apollinea* were assessed in this study through qualitative and quantitative assays of the phytochemicals and the proximate composition and minerals content from this plant.

Phenolics are important plant metabolite playing a remarkable protective role against several health disorders [30]. Phenolics possess various biological activities, for different types of ailments [31]. There are scientific reports showing relationship between phenolic content and antioxidative activity of the methanolic extract of different plants. In this study also, we can correlate the antioxidative activity with the presence of phenolics and other compounds. Presence of phenolics is one of the mechanisms of the overall antioxidant activities in plant samples and is mainly due to their redox properties [32].

Terpenoids exhibit pharmacological activities like anti-inflammatory, anticancer, antihyperglycemic, antipasmodic anti-malarial, inhibition of cholesterol synthesis, anti-viral and anti-bacterial activities [33]. Additionally, terpenoids can be used as protective substances in storing agriculture products as they are known to have insecticidal properties as well [34]. Due to the presence of gallic and diagallic acids, tannins have the oxidation inhibiting activity [35]. Flavonoids, on the other hand are potent water-soluble antioxidants and prevent oxidative cell damage, with considerable anticancer activity [36]. It also helps in managing diabetes induced oxidative stress. Here in this study, we report significant flavonoid contents in the extract of *T. appollinea*.

The alkaloids are the class of nitrogenous compounds and a diverse array of which are produced by numerous plants as secondary metabolites [37]. They are usually produced by plants for protective functions like from stresses or defense toward herbivory or pathogenic organisms and insects [37]. They have been reported to be active against many metabolic disorders like hypertension, arrhythmia, malaria, cancer and cardiovascular problems [38]. Saponin’s natural tendency to destroy microbes makes them good candidates for treating fungal, yeast infections, and serve as natural antibiotics, which help the body to fight infections and microbial invasion [39].

The aerial parts of *T. apollinea* contains crude fibre, all fall in the range of recommended dietary allowance (RDA) for fibre in children, adults, pregnant and lactating mothers [40]. But due to the reported toxicity of this plant [41, 42], it cannot be recommended to consume as such. Crude fibre can decrease serum cholesterol levels, which is the main risk of coronary heart disease, hypertension, diabetes, colon and breast cancer [40]. Ash content is generally taken to be a measure of the mineral content of the original food. Natural food products should have a general ash content of about 5% while processed food can have ash content ranging over 10%. According to A.O. A.C, this study shows that *Tephrosia* species have acceptable levels of ash content as natural food products. Wild edible plants high in carbohydrate are helpful for the body to meet up with daily activities, and most of them contains antioxidant compounds [43]. In this plant high carbohydrate content are more advantageous than those with excess protein because the body does not require too much of protein and fat. Moisture content determination is one of the most fundamental and important analytical procedure. According to Yisa et al. [44] high moisture content increases perishability as the fruits are more susceptible to microbial infections.

Minerals such as calcium and sodium are essential in maintaining a good health. Besides that, zinc plays quite a crucial role as well. There has also been an increasing concern in the amount of minerals in food as human’s fundamental minerals [45]. In the presence, micro and macro elements are vital for the overall mental and physical well being; and are important constituent of bones, teeth, tissues, muscles, blood and nerve cell and can help in acid–base balance [46]. Deficiency of vital and trace elements in human can occur even under the most practical dietary conditions and in many diseased statuses. The aerial parts of *T. apollinea* had significantly higher potassium concentrations. Potassium content in the body was reported to increase iron utilization and beneficial to control hypertension through body fluid [47]. While the remaining nutrients like Ni, Pb and Cr had negligible concentration levels in *T. apollinea*. Cr is considered toxic even at 5 mg/l and due to this reason, most of the plants shows lesser concentration of Cr as compared to that of recommended level for toxicity in plants. The deficiency of Mg causes semi coma, diabetes mellitus and neurological disturbances [48].

The function of DPPH assay method is that the antioxidants respond with the stable free radical. During the free radical effect, DPPH (α,α-diphenyl-β-picrylhydrazyl) is converted into α,α -diphenyl- β-picrylhydrazine with colour change. The rate of colour change slowly decreases to indicate the scavenging potentials of the sample antioxidant. The methanolic extracts of *T. apollinea* contain flavonoid, saponins, tannins, phenolics and aromatic compounds. Shehab et al. [31] showed the ethanolic and methanolic extracts of herbs showed noticeable DPPH radical-scavenging activity as compared to ascorbic acid. All these bioactive compounds were able to discolour DPPH solution by their hydrogen donating ability [49]. *T. apollinea* possesses significant reducing power property in methanol extract. It means, most of the compounds derived from this plant are electron donors, and therefore it can reduce the oxidized intermediates of lipid peroxidation process, so that they can act as primary and secondary antioxidants [32]. The maximum DPPH radical scavenging activity in fresh pulp can be attributed to the richness of the total phenolic components. Energy and nutrient values of medicinal plant samples are mainly used to translate medicinal samples intakes as intakes of food components.

## Conclusion

In this study, the aerial parts of the *T. apollinea* showed considerable phytochemicals and thus the plant extract can be used as an efficient free radical scavenger. The study also demonstrated post harvest storage quality of plant material because of low level of moisture content, indicative of its prolonged shelf life. The antioxidant qualities are also significant, thus making it an excellent ingredient of traditional medicine and can be useful in the synthesis of active medicinal compound for modern medicine. However, individual active compound isolation and characterization is needed in order to elucidate the structure of phyto-active principles compounds, which could be used for pharmaceutical use, which is the next step in our study.

## Abbreviations

DPPH: 1,1-diphenyl-2-picrylhydrazyl
IC 50: 50% inhibition
ROS: reactive oxygen species
AOAC: Association of Official Analytical Chemists
GAE: gallic acid equivalent
QE: quercetin equivalents
RDA: recommended dietary allowance

## References

[1] Sakkir S, Kabshawi M, Mehairbi M (2012). Medicinal plants diversity and their conservation status in the United Arab Emirates (UAE). J Med Plants Res..

[2] Hasnah KA, Cheruth AJ, Salem MA, Maqsood S (2012). Evaluation of antioxidant activity of *Cleome brachycarpa* Vahl ex DC., an under-exploited desert plant of United Arab Emirates. Pharmacologyonline.

[3] Cheruth AJ, Al Naqbi KM, El-Kaabi AA, Odeh OW, Kandhan K, Maqsood S, Kurup SS, Sakkir S (2016). In vitro antioxidant activities and screening of phytochemicals from methanolic and ethyl acetate extracts of *Calligonum comosum* L’Her. Oriental Pharm Exp Med..

[4] Habila JD, Bello IA, Dzikwe AA, Ladan Z, Sabiu M (2011). Comparative evaluation of phytochemicals, antioxidant and antimicrobial activity of four medicinal plants native to northern Nigeria. Aust J Basic Appl Sci..

[5] Santhi R, Lakshmi G, Priyadharshini AM, Anandaraj L (2011). Phytochemical screening of *Nerium oleander* leaves and *Momordica charantia* leaves. Int Res J Pharm..

[6] Karthikeyan S, Laxmanappa Hoti S (2015). Development of fourth generation ABC inhibitors from natural products: a novel approach to overcome cancer multidrug resistance. Anti-Cancer Agents Med Chem.

[7] Krishnaiah D, Sarbatly R, Bono A (2007). Phytochemical antioxidants for health and medicine a move towards nature. Biotechnol Mol Biol Rev..

[8] Karthishwaran K, Mirunalini S (2012). Assessment of the antioxidant potential of Pergularia daemia (Forsk.) extract in vitro and in vivo experiments on hamster buccal pouch carcinogenesis. Asian Pacific J Trop Dis.

[9] Jongbloed M, Feulner G, Böer B, Western AR (2003) The comprehensive guide to the wild flowers of the United Arab Emirates. Environmental Research and Wildlife Development Agency.

[10] Hassan LE, Ahamed MB, Majid AS, Iqbal MA, Al Suede FS, Haque RA, Ismail Z, Ein OC, Majid AM (2014). Crystal structure elucidation and anticancer studies of (-)-pseudosemiglabrin: a flavanone isolated from the aerial parts of *Tephrosia apollinea*. PLoS ONE.

[11] Gulecha V, Sivakuma T (2011). Anticancer activity of *Tephrosia purpurea* and *Ficus religiosa* using MCF 7 cell lines. Asian Pacific J Trop Med..

[12] Ghazanfar SA, Al-Al-Sabahi AM (1993). Medicinal plants of northern and central Oman (Arabia). Econ Bot..

[13] Abou-Douh AM, Ito C, Toscano RA, El-Baga NY, El-Khrisy EE, Furukawa H (2005). Prenylated flavonoids from the root of Egyptian *Tephrosia apollinea*–crystal structure analysis. Zeitschrift für Naturforschung B..

[14] Hisham A, John S, Al-Shuaily W, Asai T, Fujimoto Y (2006). (+)-Apollineanin: a new flavanone from *Tephrosia apollinea*. Nat Prod Res..

[15] Touqeer S, Saeed MA, Ajaib M (2013). A review on the phytochemistry and pharmacology of genus *Tephrosia*. Phytopharmacology.

[16] Hossain H, Rahman SE, Akbar PN, Khan TA, Rahman MM, Jahan IA (2016). HPLC profiling, antioxidant and in vivo anti-inflammatory activity of the ethanol extract of *Syzygium jambos* available in Bangladesh. BMC Res Notes.

[17] Edeoga HO, Okwu DE, Mbaebie BO (2005). Phytochemical constituents of some Nigerian medicinal plants. Afr J Biotechnol.

[18] Harborne AJ (1998). Phytochemical methods a guide to modern techniques of plant analysis.

[19] Harborne JB, Baxter E, Harborne JB, Baxter H (1999). The handbook of natural flavonoids.

[20] Singleton VL, Rossi JA (1965). Colorimetry of total phenolics with phosphomolybdic-phosphotungstic acid reagents. Am J Enol Vitic.

[21] Zhishen J, Mengcheng T, Jianming W (1999). The determination of flavonoid contents in mulberry and their scavenging effects on superoxide radicals. Food Chem.

[22] A.O.A.C. Official methods of analysis, association of analytical chemists. 15th ed., Washington D. C. USA. 1990; 1121–80.

[23] Horwitz W, AOAC (1995). Official methods of analysis.

[24] Dastagir G, Hussain F, Khattak F (2013). Proximate analysis of plants of family Zygophyllaceae and Euphorbiaceae during winter. Sarhad J Agric..

[25] Van Soest PV, Robertson JB, Lewis BA (1991). Methods for dietary fiber, neutral detergent fiber, and nonstarch polysaccharides in relation to animal nutrition. J Dairy Sci.

[26] Al Ameri SA, Al Shaibani FY, Cheruth AJ, Al-Awad AI, Al-Yafei MA, Karthishwaran K, Kurup SS (2014). Comparative phytochemical analysis of *Moringa oleifera* and *Moringa peregrina*. Pharmacologyonline.

[27] Burits M, Bucar F (2000). Antioxidant activity of *Nigella sativa* essential oil. Phytother Res..

[28] Oyaizu M (1986). Studies on products of browning reaction–antioxidative activities of products of browning reaction prepared from glucosamine. Jpn J Nutr..

[29] Ncube NS, Afolayan AJ, Okoh AI (2008). Assessment techniques of antimicrobial properties of natural compounds of plant origin: current methods and future trends. Afr J Biotechnol.

[30] Hung TM, Na M, Thuong PT, Su ND, Sok D, Song KS, Seong YH, Bae K (2006). Antioxidant activity of caffeoyl quinic acid derivatives from the roots of *Dipsacus asper* Wall. J Ethnopharmacol.

[31] Shehab NG, Abu-Gharbieh E, Bayoumi FA (2015). Impact of phenolic composition on hepatoprotective and antioxidant effects of four desert medicinal plants. BMC Comp Alter Med..

[32] Deighton N, Brennan R, Finn C, Davies HV (2000). Antioxidant properties of domesticated and wild *Rubus* species. J Sci Food Agric.

[33] Mahato SB, Sen S (1997). Advances in triterpenoid research, 1990–1994. Phytochemistry.

[34] Ajikumar PK, Tyo K, Carlsen S, Mucha O, Phon TH, Stephanopoulos G (2008). Terpenoids: opportunities for biosynthesis of natural product drugs using engineered microorganisms. Mol Pharm.

[35] Ihekoronye AI, Ngoddy PO (1985). Integrated food science and technology for the tropics.

[36] Okwu DE (2004). Phytochemicals and vitamin content of indigenous spices of South Eastern Nigeria. J Sustain Agric Environ..

[37] Nascimento NC, Fett-Neto AG. Plant secondary metabolism and challenges in modifying its operation: an overview. Plant Sec Metab Eng. 2010:1–3. doi:10.1007/978-1-60761-723-5_1 10.1007/978-1-60761-723-5_1 20552440

[38] Olivier DK, Van Vuuren SF, Moteetee AN (2015). *Annickia affinis* and *A. chlorantha* (*Enantia chlorantha*)–a review of two closely related medicinal plants from tropical Africa. J Ethnopharmacol.

[39] Kang J, Zeng B, Tang S, Wang M, Han X, Zhou C, Yan Q, He Z, Liu J, Tan Z (2016). Effects of *Momordica charantia* Saponins on *In vitro* ruminal fermentation and microbial population. Asian Aust J Anim Sci..

[40] Ishida H, Suzuno H, Sugiyama N, Innami S, Tadokoro T, Maekawa A (2000). Nutritive evaluation on chemical components of leaves, stalks and stems of sweet potatoes (*Ipomoea batatas* Poir). Food Chem.

[41] Nenaah GE (2014). Toxic and antifeedant activities of prenylated flavonoids isolated from *Tephrosia apollinea* L. against three major coleopteran pests of stored grains with reference to their structure–activity relationship. Nat Prod Res.

[42] Suliman HB, Wasfi IA, Adam SE (1982). The toxic effects of *Tephrosia apollinea* on goats. J Comp Pathol.

[43] Avasthi AS, Bhatnagar M, Sarkar N, Kitchlu S, Ghosal S (2016). Bioassay guided screening, optimization and characterization of antioxidant compounds from high altitude wild edible plants of Ladakh. J Food Sci Technol..

[44] Yisa J, Egila JN, Darlinton AO (2010). Chemical composition of *Annona senegalensis* from Nupe land, Nigeria. Afr J Biotechnol..

[45] Arslan D, Özcan MM (2008). Evaluation of drying methods with respect to drying kinetics, mineral content and colour characteristics of rosemary leaves. Energy Conv Manag..

[46] Uwangbaoje LO (2012). The mineral and phytochemical analysis of the leaves of *Senna alata* and *Cajanus cajan* and their medicinal value. Int J Biol Pharm Allied Sci..

[47] Goyeneche R, Roura S, Ponce A, Vega-Gálvez A, Quispe-Fuentes I, Uribe E, Di Scala K (2015). Chemical characterization and antioxidant capacity of red radish (*Raphanus sativus* L.) leaves and roots. J Funct Foods..

[48] Adriano DC (1986). Trace elements in the terrestrial environment.

[49] Ahmed D, Chaudhary MA (2009). Medicinal and nutritional aspects of various trace metals determined in *Ajuga bracteosa*. J Appl Sci Res..

